# Measuring disability: a systematic review of the validity and reliability of the Global Activity Limitations Indicator (GALI)

**DOI:** 10.1186/s13690-018-0270-8

**Published:** 2018-05-28

**Authors:** Herman Van Oyen, Petronille Bogaert, Renata T. C. Yokota, Nicolas Berger

**Affiliations:** 1 Department of Epidemiology and Public Health, Sciensano, J. Wytsmanstraat 14, 1050 Brussels, Belgium; 2 Department of Public Health, Ghent University, De Pintelaan 185, 9000 Ghent, Belgium; 3 Department of Sociology, Interface Demography, Vrije Universiteit Brussel, Pleinlaan 2, 1050 Brussels, Belgium; 4 Department of Social & Environmental Health Research, London School of Hygiene & Tropical Medicine, Keppel Street, London, WC1E 7HT UK

**Keywords:** Disability, Participation restriction, Healthy life years, Validity, Reliability, Summary measure of population health, GALI

## Abstract

**Background:**

GALI or Global Activity Limitation Indicator is a global survey instrument measuring participation restriction. GALI is the measure underlying the European indicator Healthy Life Years (HLY). Gali has a substantial policy use within the EU and its Member States. The objective of current paper is to bring together what is known from published manuscripts on the validity and the reliability of GALI.

**Methods:**

Following the PRISMA guidelines, two search strategies (PUBMED, Google Scholar) were combined to identify manuscripts published in English with publication date 2000 or beyond. Articles were classified as reliability studies, concurrent or predictive validity studies, in national or international populations.

**Results:**

Four cross-sectional studies (of which 2 international) studied how GALI relates to other health measures (concurrent validity). A dose-response effect by GALI severity level on the association with the other health status measures was observed in the national studies. The 2 international studies (SHARE, EHIS) concluded that the odds of reporting participation restriction was higher in subjects with self-reported or observed functional limitations. In SHARE, the size of the Odds Ratio’s (ORs) in the different countries was homogeneous, while in EHIS the size of the ORs varied more strongly. For the predictive validity, subjects were followed over time (4 studies of which one international). GALI proved, both in national and international data, to be a consistent predictor of future health outcomes both in terms of mortality and health care expenditure. As predictors of mortality, the two distinct health concepts, self-rated health and GALI, acted independently and complementary of each other. The one reliability study identified reported a sufficient reliability of GALI.

**Conclusion:**

GALI as inclusive one question instrument fits all conceptual characteristics specified for a global measure on participation restriction. In none of the studies, included in the review, there was evidence of a failing validity. The review shows that GALI has a good and sufficient concurrent and predictive validity, and reliability.

## Introduction

Ageing of populations defies health and social policies. Population ill-health and especially disability are major challenges as there is currently no consistent evidence that the lengthening of life expectancy goes with a reduction in the total lifetime days of disability, the so-called compression of morbidity [1].

The concept of disability is complex and multidimensional. In initial medical models, disability was viewed as a problem residing solely in the persons affected. Disability referred to consequences of chronic or acute diseases or accidents on the functioning of specific body systems and on mental, physical and sensory functions in terms of (1) impairment or dysfunctions and structural abnormalities in specific body systems; (2) disability or restrictions in basic physical and mental actions and (3) handicaps or difficulties in doing activities of daily life [2–5]. More biophysical-social models introduce the person-environment perspective of the disablement process: disability as the outcome of the interaction of a person and his environment [6] and the dynamics of disability which is affected by how a person’s capacity fits the environmental demand and results in participation [3]. Participation restriction is defined as limitations in the performance of roles and social involvement in different settings such as work and employment, school, leisure, parenting, housework, community, social and civic life [7]. Because participation is influenced by environmental factors and social norms, any measure of participation restriction cannot differentiate the impact of the impairment and functional limitations from the impact of accommodations and enabling environments [8, 9].

Disability can occur in any human activity and settings. Adding to this complexity, instruments measuring disability differ in the domains of functioning included, in their goals to measure either capacity (without any personal or equipment assistance) or performance (with assistance), or to measure also disability symptoms (pain, weakness, endurance, …), levels of severity or the duration of the disability. Traditional survey instruments measure a limited number of tasks (5 to 7) in the domain of personal care (ADL (Activity of Daily Living)) or in the domain of household management (IADL (Instrumental ADL)). Other survey instruments have either increased the number of disability questions by adding more and more tasks [10] or have developed short set of disability questions that have good coverage of activities [11, 12]. At the same time there has been a quest to measure disability with parsimony similar to the parsimony in measuring health using the global one-item survey instrument on self-rated health (SRH) [13, 14].

In response to the call for parsimony, a global survey instrument to measure disability, the Global Activity Limitation Indicator or GALI, was proposed. The development of GALI occurred in the framework of the creation of a coherent set of indicators to monitor health across Europe [15]. GALI was part of set of 10 survey instruments including three global one-item survey instruments were proposed covering distinct health concepts: perceived health, chronic morbidity and participation restriction [16]. The 3 global questions define the Minimum European Health Module (MEHM) [17]. At the time of the development of the GALI, the beta version on the International classification of Functioning, Disability and Health (ICF) served as conceptual framework [6, 18, 19]. Because of its implicit reference to the ability for societal participation in a variety of non-specified settings and non-specified domains of life (such as employment, school, housework, and leisure) using the wording “activities people usually do”, GALI is intended to be a global self-reported measure of participation restriction. Additional conceptual criteria were the health relatedness of the cause of disability, the generic normative comparison in the level of participation, the long-standing duration of the disability (a duration of at least 6 months) and the ability to measure levels of severity [15, 18]. To accommodate the results of cognitive testing in relation to the severity options in the answer categories, the wording “to what extent” was added in the final version [20]:

“For at least the past 6 months, to what extent have you been limited because of a health problem in activities people usually do?”

Would you say you have been: s*everely limited,*

*limited but not severely, or*

*not limited at all?*

Being part of the MEHM, GALI is used in major European health and non-health surveys such as the European Health Interview Survey (EHIS), Survey on Income and Living Conditions (SILC) and the Survey of Health, Ageing and Retirement in Europe (SHARE). Since 2004, GALI is also the underlying measure of the European indicator “Healthy Life Years (HLY)”. HLY is a measure of disability free life expectancy and was presented in the set of structural indicators selected and defined to help measure progress in strategic European policies such as the 2000 Lisbon strategy and the European 2020 strategy on Active and Healthy Ageing [21]. HLY is one of the components of the Active Ageing Index [22]. At the national level, countries such as France, have selected HLY as one of their high level indicators for long term evaluation of their economic, social and environmental policies [23]. GALI also fits the requirement to follow-up European and United Nations disability policies, that stress the importance of full and effective participation as main policy outcome [24, 25]. More recently, GALI, as underlying health measure of HLY, contributes to the scoreboard indicators of the European Pillar of Social Rights [26] . Due to its high informational value, its relative simplicity and its compliance elaborated by the European Union, HLY has been proposed to be the instrument in designing social security solutions [27].

Given the use of GALI within the European Union, especially the fact that it is the measure underlying the European indicator HLY, the objective of current paper is to bring together what is known from published manuscripts on the validity and/or the reliability of GALI. Construct validity evaluation has been divided into translation validity, a more qualitative process and criterion validity, a quantitative approach [28]. Current review focus on the quantitative validity, as translation validity is linked to the conceptual criteria used for the GALI development [9, 15].

## Methods

**Fig. 1:**
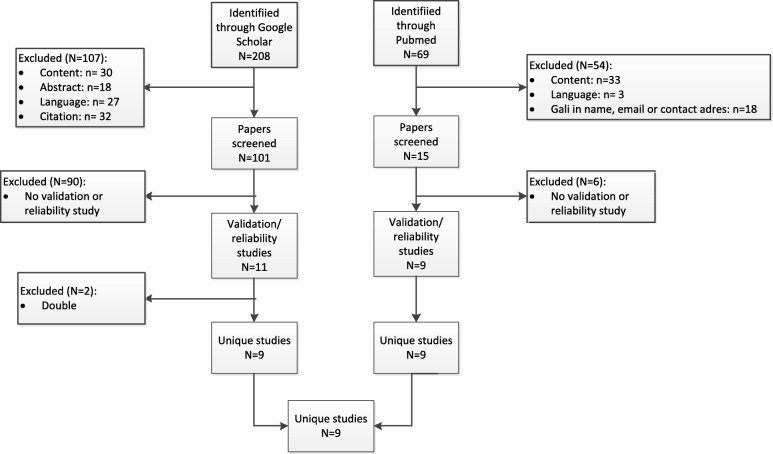
PRISMA flow chart [29]: validation and reliability studies of the Global Activity Limitation Indicator (GALI) selection, 2000–2017

Two search strategies were combined by HVO and NB in January 2017 to identify peer reviewed manuscripts published in English with publication date 2000 (the period GALI was developed) or beyond. Following the PRISMA guidelines, manuscripts were independently evaluated by HVO and NB first on the titles and abstracts and in a second stage on the text. The result of the search and manuscript selection is summarized in a PRISMA flow chart (Fig. 1) [29]. First, PUBMED database (https://www.ncbi.nlm.nih.gov/pubmed) was used with {“global activity limitation”[All Fields] OR (GALI[All Fields] NOT GALI[Author])} as search protocol. Of the 69 publications 54 were excluded: content not related to the topic (e.g. Gali as part of a name of a butterfly “Calisto franciscoi Gali”), language other than English, Gali in name, email or contact address of one of the authors). Of the remaining 15 articles, 9 articles were retained as manuscripts studying the validity or reliability of GALI. A second search used the Google Scholar database (https://scholar.google.com/) using as search protocol “global activity limitation”. Of the 208 references 107 were excluded (content not related to the topic, language other than English, only an abstract, and citations). The remaining 101 articles were screened to identify manuscripts published with the objective to estimate the validation or reliability of the GALI (*N* = 11). Double publications were excluded (*N* = 2). The double publications were a result of the publication of institutional working papers or reports prior to the publication of a manuscript in a scientific journal. All 9 manuscripts retained were found in both databases. Manuscripts were grouped as reliability studies, concurrent validity studies (cross-sectional studies measuring an association) or predictive validity studies (ability of GALI to predict an outcome) [28]. We further distinguished between national and international studies, as international studies may be more sensitive to total survey error due, for example, to the lack of international harmonization at different stages of the study [30].

## Results

The classification of the manuscripts by type of study is given in Table 1.

**Table 1:** Studies estimating the validity or reliability of the Global Activity Limitation Indicator (GALI) by study design, period 2000–2017

Reference	Study setting and population	Health outcome used in the comparison with the GALI	Severity level GALI	Statistical measure	Key findings
Concurrent validity studies					
Van Oyen et al. 2006 [32]	National HIS* 2001 Belgium Population aged 15+ *N* = 9168	Self-reported: ADL*, SF-36* physical domain score, number of self-reported chronic physical conditions out of a list of 29 conditions, number of mental conditions (depression, anxiety, somatization, sleep disorders) from the SCL-90R*, GHQ-12* scale for mental wellbeing and CMI*	Yes	Predicted probability distribution of GALI distribution, POR* from proportional odds models, heterogeneity across demographic variables	• The probability distribution of GALI by severity level fits appropriately against indicators measuring mental and physical illness both in subjects with or without ADL limitations; • 95% of subjects without ADL limitations and no mental or physical health problems do not report participation restrictions; • Subjects with ADL limitations report participation restrictions and the severity level of participation restriction is higher in function of the level of severity and the number ADL limitations and there is no evidence for heterogeneity across gender, age, education and language; • The probability distribution of GALI by severity level is associated with the different physical and mental morbidity measures; A dose-response relationship is observed; • The measures of associations are not as strong for mental health problems compared to physical health problems.
Cabrero-Garcia et al. 2014 [31]	National HIS 2006 Spain Population aged 65+ *N* = 7835	Self-reported: physical and mental morbidity, functional disability (ADL, IADL and mobility) Concurrent comparison of the associations of GALI and the association of SRH* with the health outcomes: FCI*, GHQ-12 scale for mental wellbeing, Functional disability*	Yes	Spearman correlation, predicted probability of GALI from fractional polynomial models, MOR* from multinomial logistical regression	• GALI is primarily a measure of functional status and is secondarily associated with physical and mental morbidity whereas for SRH physical morbidity and to a lesser extend mental morbidity are the main correlates; • The odds of having (severe) participation restrictions increased with the level of functional limitations (number), the physical and mental morbidity, suggesting a dose-response relationship; • Mental morbidity was as strong a correlate of GALI as of SRH, whereas physical morbidity was less strong a correlate of GALI compared to SRH.
Jagger et al. 2010 [34]	International SHARE* 2004 11 EU countries Population aged 50+ *N* = 27340	Measurement: maximum grip strength and walking speed (in subjects aged 75+) Self-reported: ADL, IADL, and walking limitations	No	Predicted probability distribution of GALI, ORs from logistic regression models, Random-effects meta-analysis to assess heterogeneity of associations between countries	• GALI effectively capture disability as measured by both the self-reported as objective measures of functional limitations; • The likelihood of reporting participation restrictions increases as the severity of functional limitations increases in both the self-reported as objective measures of functional limitations; • The likelihood of reporting no participation restriction in subjects with limitations is non-zero, though small and a minimum for the most severe measure, the ADL; • Cross-country comparison did not provide any evidence for heterogeneity for the OR of having participation restrictions in function of the self-reported ADL and the objective measures’; • In all countries, the odds of having participation restrictions was higher in subjects with IADL limitations. The size of the effect was however more pronounced in some countries compared to others.
Berger et al. 2015 [33]	International EHIS* 2007–2010 14 EU countries Population aged 15+ *N* = 152,796	Self-reported: ADL, IADL and functional limitations	No	Predicted probability distribution of GALI, ORs from logistic regression models, Random-effects meta-analysis to assess heterogeneity of associations between countries	• GALI is significantly associated with ADL and IADL limitations and functional limitations; • The likelihood of reporting participation restrictions increases as the number of ADL and IADL limitations and the severity of functional limitations increased; • The likelihood of not reporting participation restrictions decreases as the number of ADL and IADL limitations and the severity of functional limitations increased; • In all countries, the odds of having participation restrictions was higher in subjects with ADL, IADL and functional limitations. The size of the effect was more pronounced in some countries compared to others providing evidence for heterogeneity of the effect size.
Predictive validity studies					
Berger et al. 2015 [36]	National HIS 2001 linked with mortality and migration database (National Register), 2001–2010 Belgium Population aged 15+ *N* = 8583, 902 deaths	Mortality (follow-up to 10 years)	Yes	MRRs* from Poisson regression models; Comparison of relative predictive ability of GALI compared to SRH*	• Compared to individuals without participation restrictions, subjects with moderate or severe participation restriction have a 1.8 to 3.0 increased mortality rate over the 10 years of follow-up; • The effect does not vary significantly by gender, education or age, except in subjects under age of 50 years; • SRH and GALI are complementary predictors of mortality, with some indications of a stronger effect of SRH; • The predictive effect of SRH and GALI slightly decrease over time.
Van der Heyden et al. 2015 [37]	National HIS 2008 linked with mortality within the Health Insurance database, 2008–2010 Belgium Population aged 65+ *N* = 1894, 178 deaths	Mortality (follow-up to 2 years)	No	MRRs from Poisson regression models; Comparison of relative predictive ability of GALI compared to SRH	• Subjects with participation restriction have a 2.4 increased mortality rate over the 2 years of follow-up; • The effect does not vary by gender; • In men, SRH and GALI are complementary predictors of mortality, whereas in women this is only so for GALI.
Van der Heyden et al. 2015 [35]	National HIS 2008 linked with Health Insurance database including expenditure in 2008–2010 Belgium Population aged 15+ *N* = 7286	Health care expenditure (Health insurance, out of-pocket, supplement)	Yes	Linear regression after logistic transformation of costs; Cost ratios were estimated to compare expenses to a reference; Decomposition of differences in expenses using the Blinder-Oaxaca method	• Moderate and severe participation restriction increases all health expenses by 3 to 6-times; • The increase is the more pronounced in the reimbursed health care expenditure; • In absence of any chronic condition, moderate and severe participation restriction increases all health care expenditure by 2.5 to 4.5 times; • Chronic conditions explain only 22% of the differences in health care expenditure by level of participation restriction.
Verropoulou et al. 2015 [38]	International SHARE* 2004 with follow-up to re-interview in wave 2006 /2007 11 EU countries Population aged 50+ *N* = 17,941, 696 deaths	Mortality (follow-up 2 to 3 years)	Yes	Hazard ratios from Cox proportional hazard models; Comparison of relative predictive ability of GALI compared to SRH	• Both GALI and SRH are significant predictors of mortality in separate models; • When adjusting for specific health indicators (asthma, cancer, depression, mobility, IADL, orientation), GALI and SRH (only men) were significant but the magnitude diminished; • GALI and SRH add information on top of specific health indicators; • When GALI and SRH are included in one model, GALI was only significant in women, suggesting a partial conceptual overlap as there is a correlation between GALI and SRH; • SRH and GALI represent different aspects of health.
Reliability studies					
Cox et al. 2009 [39]	National Food Consumption Survey 1st and 2nd visit Belgium, Population aged 15+ *N* = 170	Twice self-reported GALI within time window between 11 and 55 days	Yes	Pearson correlation coefficients, weighted Kappa coefficients	• Both Pearson (0.73) and Kappa coefficient (0.68) indicate an acceptable reliability; • Agreement is significantly higher for males (Kappa = 0.82) compared to females (Kappa = 0.54); • Agreements did not differ by education level, age, time span and language (French, Dutch).

*HIS Health interview survey

*ADL* Activities of Daily Living

*SF-36* Short Form Survey

*SCL-90R* Symptoms Check List

*GHQ-12* General Health Questionnaire

*CMI* Composite Morbidity Indicator: no illness, only mental illness, only physical illness and both mental and physical illness

*POR* Proportional Odds Ratios

*MOR* Multinomial Odds Ratios

*SRH* Self-Rated Health

*FCI* Functional Comorbidity Index based on a list of 16 chronic conditions including obesity, hearing and visual impairments

Functional disability: based on a 27 items related to I/ADL and mobility

Washington group instrument: ref. = 32,350

*SHARE* Survey of Health and Retirement in Europe

2004 survey was done in Austria, Belgium, Denmark, France, Germany, Greece, Italy, the Netherlands, Spain, Sweden, Switzerland

*EHIS* European Health interview survey

2007–2010 surveys were done in Belgium, Bulgaria, Cyprus, Czech Republic, France, Greece, Hungary, Latvia, Malta, Poland, Romania, Slovakia, Slovenia, Spain

*MRR* Mortality Rate Ratio

### Concurrent validity

Concurrent validation studies are cross-sectional studies with the objective to measure how GALI relates to other health measures. As there is no gold standard, the associations are mainly measured using other health components such as chronic (co)morbidity or other dimensions of the disablement process, e.g. functional limitations in activities. Two studies were national [31, 32] and 2 were international [33, 34]. In one of the international studies, GALI could be evaluated against the results of objective measures of functional limitations [34]. The age groups included in the studies varied between subjects 15 years and older, 50 years and older or 65 years and older. One of the international studies focused on the population 15 years and older but provided, for comparison purposes, tables and graphs for the population 50 years and older as supplementary material [33]. The national studies considered GALI by severity level, while the international studies ignored the severity level.

Van Oyen et al. used the 2001 Belgian Health Interview survey [32] to evaluate GALI against (1) Activities of Daily Living (ADL) limitations (categorical by level of severity, and by number of limitations); (2) the Short Form Survey (SF-36) physical domain score; (3) the number of self-reported chronic physical conditions out of a list of 29; (4) the number of mental conditions (depression, anxiety, somatization, sleep disorders) based on 4 subscales of the Symptoms Check List (SCL-90R) and (5) a mental well-being score using the General Health Questionnaire (GHQ-12). A composite morbidity indicator (CMI: categorized as no illness, only mental, only physical illness and both) was used to measure the associations stratified by the ADL functional limitation status. The results indicated that all health indicators were positively associated with GALI. The participation restriction distribution by severity level was positively associated with both the number and severity of ADL limitations, the SF-36 physical domain score, the number of chronic conditions and the mental health indicators (mental health comorbidity score or the GHQ-12). E.g., without any ADL limitations the predicted probability distribution of being without, with mild or with severe participation restriction was respectively 0.82, 0.15, 0.03 compared to 0.20, 0.43, 0.37 and 0.13, 0.38, 0.49 in people with at least one ADL limitation or with at least one severe ADL limitation. When people were limited in 6 ADLs, the GALI probabilities of reporting no, mild and severe restrictions were respectively 0.03, 0.10 and 0.87. Using the GHQ-12 mental well-being score, the predicted probabilities of no, mild and severe GALI restrictions changed from respectively 0.82, 0.14, 0.04 (best GHQ-12 score) to 0.36 0.36 0.28 (worst GHQ-12 score). The CMI was associated with participation restriction both in people with and without ADL limitations. In the population free of ADL limitations, and compared to subjects reporting non illness, the participation restriction prevalence and especially the prevalence of being severely restricted increased gradually in people reporting only mental illness, reporting only physical illness or reporting both mental and physical illness. A similar trend was observed in subjects with ADL limitations but the prevalence of participation restriction and severe restriction was substantially higher within each morbidity level. E.g., the predicted probability of no participation restrictions in subjects without ADL limitations and without any mental or physical illness was 0.95 and dropped to respectively 0.90, 0.80 and 0.57 in subjects reporting only mental illness, only physical illness or both; in people with ADL limitations the predicted probabilities of no participation restriction were respectively 0.46, 0.43, 0.37 and 0.12.

The second concurrent validation study used the 2006 Spanish National Health Survey but included only subjects 65 years and older to test (1) if GALI is primarily correlated with functional disability and secondarily with morbidity, and (2) if Self Rated Health (SRH), in contrast, is primarily correlated with morbidity and secondarily with functional disability [31]. Associations were sought with a functional comorbidity indicator (FCI) based on a list of 16 chronic conditions including obesity, hearing and visual impairments, the GHQ-12 for mental ill-health and a functional disability measure (based on 27 items related to IADL/ADL and mobility). The Spearman correlation coefficients of FCI, GHQ-12 and functional disability were 0.35, 0.45 and 0.58 with GALI compared to 0.46, 0.44 and 0.36 with SRH. The predicted probability of participation restriction indicated a greater effect in function of the number of functional disabilities compared to the comorbidity indicator while the inverse was observed for the predictive probability of not being in very good/good SRH. The predicted probabilities for GALI and SRH were similar in function of the GHQ-12. Compared to subjects with no functional disability (ADL/IADL or mobility), the multinomial odds ratios (MORs) of participation restriction and severe participation restriction were respectively 1.44 and 2.02 in subjects with one functional disability (ADL/IADL or mobility) and respectively 8.94 and 64.84 when limitations in 11 functions were reported. The MORs for participation restriction and severe participation restriction were respectively 1.96 and 2.00 for subjects with a FCI score of 1 and respectively 7.49 and 7.96 for people with a FCI score of 7. The MORs of having participation restriction and severe participation restriction in people with a GHQ-12 score of 1 and of 7 were respectively 1.32 and 1.61; and 3.42 and 8.05. The MORs of fair SRH and very poor/poor SRH indicated a similar pattern but were more extreme in function of the functional comorbidity score while less extreme in function of the functional disability indicator. These results suggest that GALI was primarily a measure of functional status and secondarily a measure of physical and mental morbidity whereas for SRH, physical morbidity and to a lesser extend mental morbidity were the main correlates.

The two concurrent international studies [33, 34] followed a similar statistical analysis plan using the data from SHARE and EHIS: the estimation of the predicted GALI probability distribution by fitting logistic regression and random-effects meta-analysis models to evaluate heterogeneity of the association between countries (Table 1). The 2004 SHARE study covered 11 EU countries [34], while the EHIS (2007–2010) used data from 14 EU countries [33]. Main differences are related to the age groups included, the use of observed measures and the stronger overall survey design homogeneity between countries in the SHARE survey compared to the EHIS survey design. In both studies, the severity level information of GALI was ignored in the analysis. The GALI was evaluated against the number of ADL and IADL limitations. The EHIS data also provided an evaluation against a function limitation measure by severity based on the self-reported moderate or severe problems in at least one of the following 6 functions: walking a certain distance, going up and down the stairs, carrying in the hands or arms, using hands and fingers to manipulate small objects, biting and chewing. The objective instruments to evaluate the validity of the GALI in SHARE measured the hand grip strength and the walking speed (in 75 years or older only; reported walking limitations in those 50–74 years). In the SHARE study, the probability of the reported participation restriction was lower when the grip strength or walking speed were higher. Similarly, and in both studies, the probability of having participation restriction was higher as the number of ADL or IADL limitations increased or if the level of severity of those limitations increased (EHIS only). In all countries and in both studies, the direction of the association, measured as ORs, between GALI and the other health measures was as expected. That is: the odds of reporting participation restriction is higher in subjects with poorer functioning and disability measures (either in function of the ADL, IADL, the physical functional limitations or in function of low hand grip strength or walking speed). In the population 50 years and over, the overall random effect meta-regression ORs were less extreme in the SHARE study compared to the EHIS: e.g. comparing subjects with at least one ADL-limitation vs. those with none, the combined OR of having participation restriction were 8.3 in SHARE and 12.3 in EHIS; with respect to IADL limitations, the combined ORs were respectively 6.4 and 9.1. In the SHARE study, there was no significant country variation in the ORs in function of ADL, hand grip strength and walking speed. For IADL in the SHARE and for all functions and disability measures in the EHIS, the OR of having participation restriction was more pronounced in some countries compared to others, providing evidence of heterogeneity in the effect size.

### Predictive validity

The predictive validity of GALI was reported by 4 studies (Table 1). One study evaluated the predictive effect on health care expenditure in Belgium [35]; the other 3 studies focused on mortality. Two of the mortality follow-up studies used Belgian Health Interview Surveys as baseline (HIS-2001 [36], HIS-2008 [37]) while the other used the international SHARE study, wave 2004 and wave 2006/2007 [38]. The duration of mortality follow-up ranged from 2 to 3 years [37, 38] to 10 years [36]. Two of the 3 mortality studies used GALI by severity level [36, 38]. The age considered ranged from 15 years and older [36] to 50 years and older [38] and 65 years-plus [37]. In all 3 mortality studies, the predictive capacity of GALI was set off against the predictive capacity of SRH. The 3 studies identified GALI and SRH as complementary predictors of mortality, indicating that GALI and SRH share some traits but add different dimensions: health and disability. In the two studies with a short follow-up period [37, 38], adjusting for age, education and life style (SHARE only: physical activity, smoking, BMI) both GALI and SRH were significant predictors of mortality: having participation restrictions doubled (mild) and tripled (severe) the mortality rate in SHARE, while the mortality rate increased by a factor of 2.4 when no severity level was accounted for in the Belgian study [37]. In the Belgian study [37], when both GALI and SRH were included in the Poisson regression model, GALI remained a significant predictor of mortality next to SRH in both males and females, while SRH remained only significant in males. In the SHARE follow-up [38], the fully-adjusted proportional hazard model, controlling for specific morbidity indicators (asthma, cancer, depression) and disability measures (mobility, IADL), GALI and SRH, showed that SRH remained significantly associated with mortality only in men while GALI remained significantly associated with mortality only in women. Over a 10-year follow-up period [36], GALI as well as SRH were strong predictors of mortality. Adjusting for age, gender and socio-economic position, people with mild and severe participation restriction, compared to no participation restriction, had mortality rate ratio’s (MRR’s) of respectively 1.8 and 3.0. Compared to good/very good SRH the MRR in subjects with fair and bad/very bad SRH the MRR was respectively 1.8 and 3.6. When including GALI and SRH in the model, both remained significant predictors of mortality: MRR for GALI were: 1.4 (mild) and 1.8 (severe); and MRR for SRH were: 1.5 (fair) and 2.5 (bad/very bad). The predictive ability did not change with gender or socio-economic position. However, in older subjects, the predictive ability of SRH was not as strong. The impact on mortality of both GALI and SRH decreased over time but remained statistical significant in truncated follow-up periods: 0–3 years, 3–6 years and 6–10 years.

Van der Heyden et al. evaluated how GALI predicted health care expenditure using data linkage between each participant to the 2008 Belgian Health Interview Survey and the national health insurance data for the 12 months following the date of the interview [35]. Participation restriction was a strong determinant of the total health care expenditure: e.g. the population with participation restriction (21% of the population) accounted for 49% of the total health expenditure; for severe restriction (5% of the population) this was 17% of the total expenditure. The association was stronger for the reimbursed health care cost compared to the out-of-pocket payments. In subjects with no chronic conditions compared to people without participation restriction, the cost ratio of the reimbursed cost in subjects with mild or severe participation restriction was respectively 2.5 and 4.2. In people with one chronic condition or in people with ≥2 chronic conditions the cost ratio compared to no participation restriction were respectively 1.5 and 1.7 in subjects with mild participation restriction and 2.4 and 3.2 in people with severe participation restrictions. The authors decomposed the health expenditure gap between people with and without participation restriction: differences in the age distribution (20%) and in the prevalence of chronic diseases (22%) between the two groups were the main contributors to the explained differences (48%). Next to the confounding effect of age, the decomposition analysis also indicated that the impact of age on health expenditure differed by GALI severity level, suggesting an interaction effect of age. However, in the unexplained component, the coefficient of chronic conditions did not differ significantly between GALI categories, suggesting that chronic conditions in people with participation restrictions do not result in significant different health care expenditure compared to people without participation restrictions.

### Reliability

Only one study evaluated the reliability of GALI as part of the evaluation of the MEHM [39]. The study used the Belgian 2004 Food Consumption Survey in which people were visited twice at home by the interviewers. The interquartile range between the 2 visits was 17–26 days with median time of 20 days. Both the Pearson correlation (0.73) and the weighted Kappa coefficient (0.68) indicated an acceptable reliability. The stratification by gender showed a higher Kappa coefficient among males (0.82) compared to females (0.54). The agreement did not statistically differ by age (15–64 vs. 65+), by education (technical secondary or less vs. general secondary or higher), by language (Dutch vs. French) or time span between the interviews (≤20 days vs. > 20 days).

## Discussion

Defining disability is not easy. Because it interweaves medical and social domains [40], the concept of disability has led to divergent interpretations and uses [41]. People may experience disability due to health in any human activity; yet, activities included in traditional instruments with focus on ADL and/or IADL cover only a fraction of all activities [12]. The challenges of measuring disability have been tackled by two distinct approaches. One option is to include more activities, more specific answer categories, more aspects such as disability symptoms and disability in more specific settings of life [10, 42, 43]. This option induces increased respondents burden, increased survey cost, more complex analysis in order to provide condensed indicators for end users. The other option seeks short sets and/or a one single global instrument [11, 15]. GALI has been nominated the champion in parsimony [12], but the lack of simplicity and the high density of concepts in one single question may hamper its acceptability [9, 44]. Three different alternatives (decomposing GALI using filtered and routed questions or through omitting features such as the duration of the disability and/or the health relatedness) and the original GALI were evaluated against the short version (including 4 functional limitation questions) of the Washington Group on Disability instrument [11]. The four variants were randomly assigned to survey participants (*N* = 3009). The results, indicating a substantial higher sensitivity of GALI, no evidence for a better understanding of the simplified alternatives but possibly, a small advantage in specificity when bringing the duration of the participation restriction to subsequent questions, should be balanced against the cost of breaking an established chronological series [45]. Currently, Eurostat follows for the upcoming EHIS wave III (2018–2019), the recommendation of the EHLEIS working group on the blueprint for an internationally harmonized Summary Measure of Population Health [9]. The EHLEIS working group, including experts from the EU, Japan, USA, OECD and WHO met 3 times (2012, 2013 and 2014) in Paris and proposed that of the different components of disability, participation restriction in the first place and, in addition, functional limitations should be the main goals for internationally harmonized global measures [9]. GALI fits the six conceptual characteristics specified by the working group: 1. comprehensive content of participation; 2. measure of participation performance with current accommodation; 3. health relatedness of the cause of participation restriction; 4. normative comparison in the level of participation; 5. long-term duration of restriction; 6. measure severity of restriction in the response scale (at least three levels). As mentioned above, this comes at a cost of lack of conciseness and simplicity.

Of the 3 global questions that constitutes the MEHM [15], studies reporting on the concurrent and predictive validity and reliability of SRH have the longest history [46–48], while less evaluations have been done with respect to the global question on chronic disease [49].

In this manuscript, we summarize for the first time the current evidence of the validity of GALI including concurrent and predictive validity studies and reliability studies.

Current review has limitations. A first limitation of the review is that it only included peer-reviewed manuscripts published in English, identified using only one bibliographic database in addition to Google Scholar. Google Scholar was used to search also the grey literature. The authors were in close contact with the international research network on health expectancies and the disablement process (REVES). Members of the REVES network were invited to participate in a survey [9] with the aim to identify additional manuscripts. Secondly, all studies included rely on self-reported measures and the precision of the validity and reliability estimates relies upon accurate reporting. Thirdly, no quality related weighting was applied in describing the different manuscripts. E.g in contrast to the mortality follow-up in Belgium [36], the mortality follow-up within SHARE [38] did not use register data resulting in an under-numeration of the number of deaths. A possible effect of a selection bias on the predictive power of GALI and SRH on mortality cannot be excluded, but if any, the text of the manuscript claims it should be limited [38]. Next, the review was hampered by the methodological heterogeneity of the different studies: e.g. the association of GALI with health indicators in the two national concurrent validations studies [31, 32] was measured using different health indicators and different statistical modeling. Finally, the review was not registered.

To summarize, in none of the 9 studies included, there was evidence of a failing validity. The concurrent validity was evaluated in 4 studies. The two national concurrent validation studies indicated a dose-response effect by GALI severity level on the association with other health status measures with a somewhat weaker association related to the mental well-being score.The two international studies did not consider the GALI severity level. They concluded that the odds of reporting participation restriction were higher in subjects with self-reported or observed functional limitations. The strength of the association varied more strongly between the countries using the EHIS compared to the SHARE survey. The difference in homogeneity can in part be explained by the variation in the implementation of the EHIS, including different wording across countries [33]. International comparability of data submitted to Eurostat, including those on GALI is hampered as EU regulations does not include guidelines on the exact formulation of the questions within one and between surveys [20] nor on the data collection mode increasing the likelihood for differential total survey errors [30]. This under-valorizes the substantial efforts done by Eurostat to harmonize instruments in surveys [49]. Differences in accounting for the institutionalized population may further have affected the heterogeneity in the size of the association of the GALI with other health measures [50]. Although, without focus on validity, a recent study, using the 2013 Danish Health and Morbidity survey and 31 health-related indicators, corroborates the concurrent validity describing a trend towards poorer health and wellbeing in subjects with more participation restriction as well as a trend toward a less healthy life style or less contacts with social relations in function of more participation restrictions [51]. Using follow-up data, GALI proved both in national and international studies to be a consistent predictor of future health outcomes both in terms of mortality and health care expenditure. As predictors of mortality, the two distinct concepts - SRH and GALI - acted independently and complementary of each other. Only one reliability study was identified indicating a sufficient reliability of GALI.

## Conclusion

The strength of GALI as an inclusive one-question instrument is that it fits all conceptual characteristics specified for a global measure on participation restriction. The review indicates that current version of GALI has a good and sufficient concurrent and predictive validity and reliability.
